# Mechanical and Lattice Thermal Properties of Si-Ge Lateral Heterostructures

**DOI:** 10.3390/molecules29163823

**Published:** 2024-08-12

**Authors:** Liuhuan Zhao, Lei Huang, Ke Wang, Weihua Mu, Qiong Wu, Zhen Ma, Kai Ren

**Affiliations:** 1 School of Mechanical and Electronic Engineering, Nanjing Forestry University, Nanjing 210037, China; 2 School of Mechanical Engineering, Southeast University, Nanjing 211189, China; huanglei031800@163.com; 3 School of Automation, Xi’an University of Posts & Telecommunications, Xi’an 710121, China; 4 Wenzhou Institute, University of Chinese Academy of Sciences, Wenzhou 325000, China; 5 School of Agricultural Engineering, Jiangsu University, Zhenjiang 212013, China; 18255183270@163.com

**Keywords:** two-dimensional, lateral heterostructure, silicene–germanene, mechanical properties, thermal transport, molecular dynamics

## Abstract

Two-dimensional (2D) materials have drawn extensive attention due to their exceptional characteristics and potential uses in electronics and energy storage. This investigation employs simulations using molecular dynamics to examine the mechanical and thermal transport attributes of the 2D silicene–germanene (Si-Ge) lateral heterostructure. The pre-existing cracks of the Si-Ge lateral heterostructure are addressed with external strain. Then, the effect of vacancy defects and temperature on the mechanical attributes is also investigated. By manipulating temperature and incorporating vacancy defects and pre-fabricated cracks, the mechanical behaviors of the Si-Ge heterostructure can be significantly modulated. In order to investigate the heat transport performance of the Si-Ge lateral heterostructure, a non-equilibrium molecular dynamics approach is employed. The efficient phonon average free path is obtained as 136.09 nm and 194.34 nm, respectively, in the Si-Ge heterostructure with a zigzag and armchair interface. Our results present the design and application of thermal management devices based on the Si-Ge lateral heterostructure.

## 1. Introduction

In the current era of materials science, 2D materials [1,2,3,4] are highly significant on account of their distinctive characteristics and the advancement of methods for fabrication and characterization [5,6,7,8,9]. In particular, graphene [10], transition metal dichalcogenides (TMDs) [11,12], and hexagonal boron nitride (h-BN) [13] have been broadly explored for underlying applications in electronics, photonics, energy storage, and sensing [14,15,16]. Graphene can be used as one van der Waals Schottky heterostructure with GaN, showing the tunable Schottky contact between the p-type and n-type by interlayer coupling [17]. The MoS_2_ monolayer shows excellent light absorption characteristics, which can be further improved by boron phosphide used as a potential photocatalyst [18]. The ZrSi_2_N_4_ monolayer possesses novel hydrogen evolution reaction performances for which the Gibbs free energy is only −0.035 eV [19]. Furthermore, considerable 2D materials have been prepared in recent years such as SiP_2_ by optical reflection measurements [20], semiconducting graphene by using the quasi-equilibrium production method [21], and single-atom layer gold by chemical exfoliation [22]. All of these explain the excellent and tunable 2D material attributes for nanoelectronic devices.

The mechanical and thermal attributes of 2D materials are crucial in their applications, and the manipulating of these properties also appears to be more meaningful. The mechanical characteristics of MoS_2_ are essential in establishing the fracture strengths and strain attributes of MoS_2_/WSe_2_ heterostructures [23] and for comparing MoS_2_/WSe_2_ lateral heterostructures to graphene/h-BN, the resultant interfacial thermal conductivity of which is about one order of magnitude lower [24]. The exchange of S and Se atoms in MoS_2_/WSe_2_ can create the Janus MoSSe/WSSe [25,26,27,28], which has been reported to possess a curved interface that decreases the interfacial thermal conductivity [29]. Additionally, the MoSSe/WSSe heterostructure superlattice is investigated to demonstrate an ultra-flexible characteristic due to its curved interface. Silicene and germanene [30] are reported as 2D materials showing the honeycomb lattice construction resembling graphene. They have remarkable chemical and physical features, like elevated electron mobility [31], a customizable bandgap, and strong spin–orbit coupling, making them strong candidates for various nanoelectronic devices [32,33,34]. Recently, important investigations have been developed in the preparation of silicene and germanene, such as chemical vapor deposition [35,36] and molecular beam epitaxy [37,38], which are commonly used to create high-quality thin films of germanene and silicene on suitable substrates. Furthermore, mechanical exfoliation [39] and liquid phase exfoliation [40] methods have also been employed to obtain a few or single layers of silicene and germanene, allowing for precise control over the layer count for specific applications. Heterostructure assembly is a popular strategy to improve and regulate the thermal, optical, and mechanical attributes of 2D materials [41,42]. Thus, the development of lateral heterostructures of silicene and germanene can help regulate the properties of 2D materials and determine their potential applications in nanodevices.

For this study, the mechanical and thermal features of the silicene–germanene (Si-Ge) lateral heterostructure were analyzed using molecular dynamics (MD) simulations. The fracture strength can obviously be tuned by temperature, prefabricated cracks, and vacancy defects. Molecular dynamics (MD) simulations are also applicable for defect propagation, which successfully reproduced the formation of vacancy clusters in both β- and α-Si_3_N_4_, demonstrating its ability to predict defect clustering behavior and its impact on lattice parameters [43]. This study also investigates the thermal conductivity of silicene, revealing the dominance of longitudinal phonon modes in heat transport and the short phonon mean free path [44]. This study provides valuable insights into the role of defects in altering material properties, further highlighting the applicability of classical MD for defect systems. In addition, the Si-Ge lateral heterostructure’s heat transfer performance is addressed, and the response of thermal conductivity to size is presented. Accordingly, it is revealed that the Si-Ge lateral heterostructure can be used in excellent thermal management nanodevices.

## 2. Simulation Methods

**Figure 1:**
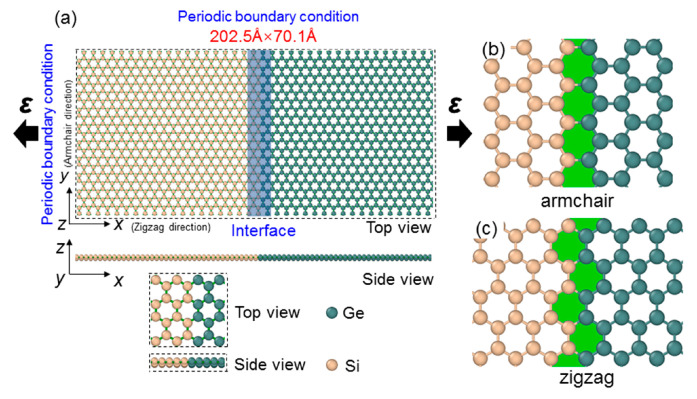
(**a**) The comprehensive construction of the Si-Ge lateral heterostructure constructed by (**c**) zigzag and armchair (**b**) configurations.

This study utilized the Large-Scale Atomic/Molecular Parallel Simulator (LAMMPS), with the aim of conducting the molecular dynamics (MD) simulations [45]. The results are visualized by the OVITO 3.10.6 software package [46]. The time step of 1.0 fs was used in the total MD simulations, which is also small enough to capture the lattice dynamics realistically used in other similar investigations [47], ensuring stable and accurate numerical values for energy conservation calculations. In order to achieve agreement between DFT calculations and experimental data, we used the Tersoff potential to feature the interplay between Si and Ge atoms [48]. In a previous study, the proposed Tersoff potential has been successfully applied to MD-based studies of silicene and germanene nanoribbons [49], thus ensuring the accuracy of the results. The model structures of silicene and germanene are shown in Figure S1 in the Supplementary Materials. The Si-Ge lateral heterostructure can be constructed with the armchair and zigzag edges along the *x* direction, named as the armchair and zigzag heterostructure, as shown in Figure 1a. The interface of the armchair and zigzag heterostructure is demonstrated in Figure 1b,c, respectively. The armchair and zigzag orientations are used in conjunction with periodic boundary conditions to create the nanosheet structure.

A Nose–Hoover thermostat maintained consistent stress and temperature during 200 ps, relaxing the entire system, and then, the surface pressure and the thermal conductivity of the studied heterostructure were investigated. All mechanical simulations were performed using a Si-Ge lateral heterostructure with dimensions approximately of 202.5 Å × 70.1 Å, with the number of Si and Ge atoms for 1000 and 1000, respectively. Before applying the external strain, the Si-Ge system was balanced at 300 K using an *NPT* ensemble for 2 ns; next the *NVE* ensemble was simulated for 2 ns. Thus, the stable Si-Ge lateral heterostructure was obtained, and then, the system underwent in-plane uniaxial stretching at a strain velocity of 2 × 10^8^ s^−1^ while maintaining zero pressure in the other directions. The temperature of the system remained constant.

In the Si-Ge heterostructure, all atoms have their respective atomic virial pressure [50].

(1) $$\sigma_{i}=\frac{1}{\Omega_{i}}\left[m_{i}v_{i}⊗v_{i}+\frac{1}{2}\underset{j\neq i}{\sum }F_{ij}⊗r_{ij}\right]$$

In the context of molecular dynamics simulations, the variables *m_i_* and *υ_i_* denote the mass and velocity vectors of atom *i*, separately, while Ω*_i_* denotes the atomic volume. The force exerted by atom *j* upon atom *i* is indicated by *F_ij_*, and *r_ij_* signifies the distance between these atomic pairs. *j* spans atoms within cut-off distance of atom *i.* According to the following equation, there are two different sources of atomic virial stress. The first term is created by thermal vibration, which is zero in static simulations, and the second by the interatomic force. Within the LAMMPS simulation framework, the stress tensor *σ_i_* is characterized by its symmetry and is composed of six distinct elements: *σ_xx_*, *σ_yy_*, *σ_zz_*, *σ_xy_*, *σ_xz_*, and *σ_yz_*. When dealing with two-dimensional (2D) materials, the contributions from *σ_zz_*, *σ_xz_*, and *σ_yz_* are typically not considered. The atomic volume is measured by separating the volume of the initially relaxed sheet from the overall atom count within the simulation system [51].

In thermal calculations, the non-equilibrium molecular dynamics (NEMD) method is used for examining the heat transport properties of the Si-Ge heterostructure. Moreover, the system is balanced at 300 K using an *NPT* ensemble, followed by relaxation with an *NVE* ensemble for 2 ns [47]. Then, the overall thermal simulations are explored for 24 ns. The heat flux (J) is computed through the energy exchange evaluation occurring between the atom with the highest thermal energy in the heat sink plate and the atom with the minimal thermal energy in the heat source plate. The heat flux (*J*) is defined as

(2) $$J=\frac{1}{V}\left[\overset{N}{\underset{i}{\sum }}\varepsilon_{i}\nu_{i}+\frac{1}{2}\overset{N}{\underset{ij,i\neq j}{\sum }}(F_{ij}\cdot \nu_{i})r_{ij}+\frac{1}{6}\overset{N}{\underset{ijk,i\neq j\neq k}{\sum }}(F_{ijk}\cdot \nu_{i})(r_{ij}+r_{ik})\right]$$

where *V* is the volume of the investigated system, *ε_i_* signifies the energy, *v_i_* stands for the velocity of atom *i*, and *r_ij_* represents the distance between atoms *i* and *j*. Forces between pairs of atoms (two-body forces) are represented as *F_ij_*, whereas interactions involving three atoms (three-body forces) are denoted by *F_ijk_*.

## 3. Results and Discussion

**Figure 2:**
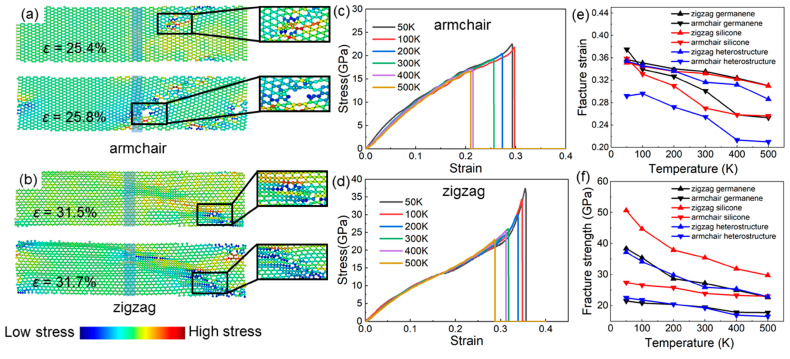
(**a**,**b**) The atomic stress contour of the initial cracking and (**c**,**d**) stress–strain behavior of the Si-Ge heterostructure with (**a**,**c**) armchair and (**b**,**d**) zigzag interfaces under external uniaxial strain. (**e**) The fracture strain and (**f**) fracture strength of the pure silicene, germanene, and Si-Ge heterostructures.

Initially, the Si-Ge heterostructure’s fracture behavior is examined at 300 K with an exterior uniaxial strain. The initial crack with the armchair and zigzag interfaces is demonstrated in Figure 2a and b, separately. One can observe that the initial cracks mainly occur in germanene, independent of the type of interface, which may be attributed to thermal fluctuations and the higher mechanical strength of silicene. The armchair heterostructure demonstrates lower fracture strains and strengths compared to the zigzag heterostructure at 300 K. More specifically, the armchair heterostructure can be subjected to about a 25.4% external strain until the initial cracks appear at 300 K, and the fracture stress is obtained at 19.28 GPa, shown in Figure 2a. As strain increases (about 25.8%), new cracks will appear in germanene. Furthermore, the obtained fracture stress of the zigzag heterostructures is 31.5%, with a strength of 25.84 GPa at 300 K, as shown in Figure 2b. Then, the further crack only extends around the initial crack with continuous strain (about 31.7%) in germanene.

Next, the temperature dependence of the stress–strain behavior of the armchair and zigzag Si-Ge heterostructures is explored, as shown in Figure 2c,d, respectively. One can see that the fracture strain strength of both the armchair and zigzag Si-Ge heterostructure can be decreased significantly as the temperature varies between 50 K and 500 K. In contrast, the hardening phenomenon is evident in the stress–strain attributes of the zigzag heterostructure when comparing it to the armchair heterostructure, which is also reported in SiC [47]. The response of the fracture strain and strength of the Si-Ge heterostructure to temperature is further explained in Figure 2e and Figure 2f, respectively. Obviously, the fracture strength of pure silicene is always higher than that of germanene when strain is applied to the zigzag and armchair orientations, which also explains why the initial crack is induced in germanene in the heterostructure. Furthermore, at varying temperatures, the fracture strength of the zigzag Si-Ge heterostructure is higher than that of the armchair one. More specifically, the fracture strength and strain of the zigzag heterostructure are obtained as 35.4% and 37.42 GPa at 50 K, while the fracture strength and strain of the armchair heterostructure are 29.2% and 22.44 GPa, respectively. With the increase in temperature up to 500 K, the fracture strain and fracture strength of the zigzag heterostructure decrease by 19.2% and 38.4%, separately. The fracture strain and strength of the armchair heterostructure are decreased by 28.1% and 26.3%, respectively. The decrease in the fracture strength of the Si-Ge heterostructure is mainly due to the high temperature-induced softening phenomenon of the material when some of the chemical bonds reach critical bond lengths and fracture [52]. The fracture strength of the Si-Ge heterostructure is smaller than that of SiC (73–78 GPa) and GeC (63–71 GPa), while it is larger than that of the MoS_2_-WSe_2_ lateral heterostructure (9–16 GPa) and the MoS_2_ monolayer (11–15 GPa). The fracture strain of the Si-Ge heterostructure is also larger than that of the MoS_2_-WSe_2_ lateral heterostructure (0.08–0.13) and smaller than that of SiC (0.36–0.44) and GeC (0.39–0.47) monolayers [23,47]. This result highlights the significant influence of temperature upon fracture attributes of Si-Ge lateral heterostructures, which provides an essential reference for designing and applying related materials.

**Figure 3:**
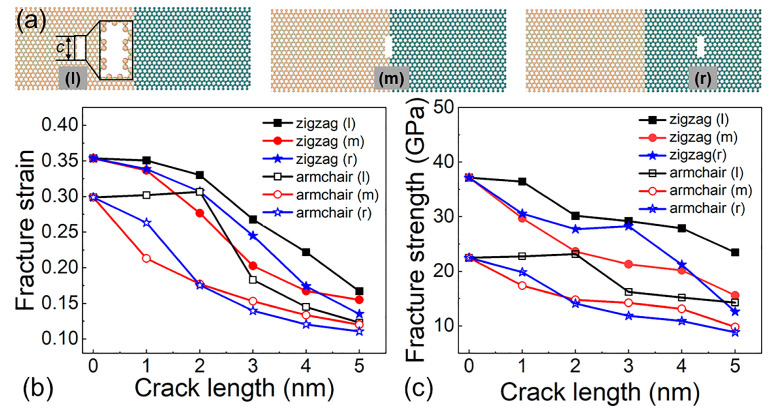
(**a**) The structure of the pre-cracked heterostructure, the tunable (**b**) fracture strength and (**c**) fracture strain of the Si-Ge heterostructure with different pre-cracked structures.

Defects are unavoidable during the fabrication of 2D materials. However, the manipulation of defects is a widely employed strategy for adjusting the properties of two-dimensional materials [53,54]. Thus, an additional investigation is undertaken to determine how pre-existing fractures affect the Si-Ge lateral heterostructure’s fracture strength and strain. As shown in Figure 3a, three defect types are considered by the locations of the cracks in silicene, at the interface, and in germanene, named as (l), (m), and (r), respectively. The width of the cracks is kept at 0.8 nm, with the length ranging from 1 to 5 nm at 50 K. When the crack type is (l), for crack lengths of 2 nm and below, the impact of an established crack on the fracture strength and fracture strain is minimal. When the crack length is minimal, silicene with cracks is still stronger than germanene without cracks, and thus, failure occurs in the germanene region. Furthermore, the continuous increased crack length presents a significant effect on the fracture strength and fracture strain, and the regions of failure shift to the silicene regions, as shown in Figure 3b,c. In the zigzag heterostructure, the failure locations all occur in the region near the pre-crack, and as crack length increases, both the fracture strain and fracture strength exhibit a decline.

**Figure 4:**
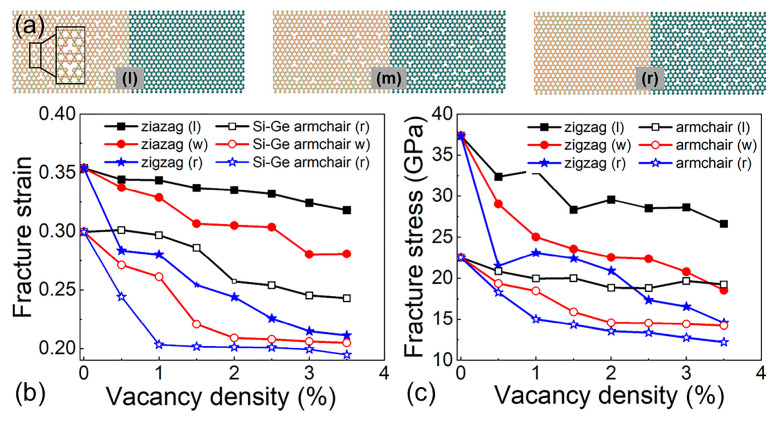
(**a**) The structure of the pre-cracked heterostructure. The tunable (**b**) fracture strength and (**c**) fracture strain of the Si-Ge heterostructure with different vacancy densities.

The response of the mechanical properties of the Si-Ge lateral heterostructure to the vacancy defect is further explored. The vacancy defect can be introduced in the silicene, overall heterostructure, and germanene, named as (l), (m), and (r), respectively, shown in Figure 4a. The fracture strength and strain of Si-Ge heterostructures with different vacancy densities at 50 K are demonstrated in Figure 4b and Figure 4c, respectively. One may note that the fracture strain and strength of the heterostructures decrease with the rising vacancy density. The germanene region is susceptible to vacancy density and decreases sharply with the increased vacancy density. We also found that when the vacancy is located in the silicene in the armchair heterostructure at less than 1.5%, the fracture is induced in the germanene, which is a very similar phenomenon to that in the above prefabricated crack investigation. Moreover, the zigzag heterostructure at the three vacancy densities (0.5% to 3.5%) showed a reduction in fracture strain of 10.2% (l), 40.3% (r), and 20.7% (w), and a reduction in fracture strength of 29.7% (l), 60.9% (r), and 50.3% (w), respectively. Similarly to the armchair heterostructure, the fracture strain decreased by 18.9% (l), 35.1% (r), and 31.6% (w), and the fracture strength decreased by 14.5% (l), 45.8% (r), and 36.9% (w), respectively. The results indicate that the vacancy defects have potential applications in modulating the mechanical attributes of the Si-Ge lateral heterostructure.

**Figure 5:**
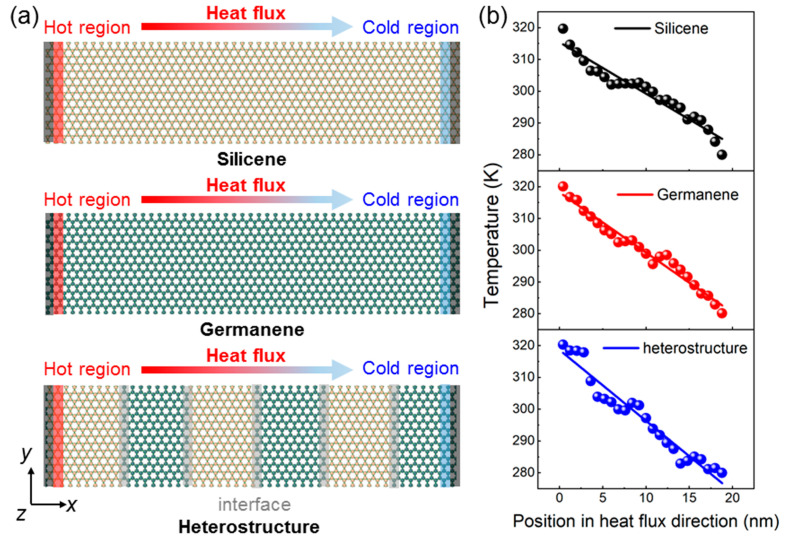
(**a**) The schematic models of pure silicene, germanene, and the lateral heterostructure used in NEMD simulations. (**b**) The obtained temperature distribution along the heat flux orientation of the system simulated.

Furthermore, exploring the thermal properties of Si-Ge lateral heterostructures is crucial for advanced thermoelectric and electronic devices. The thermal heat transport attributes of silicene, germanene, and the lateral heterostructure were examined using the NEMD method. Figure 5a shows the simulation models in which the left and right ends of silicene, germanene, and the lateral heterostructure are fixed and the cold (280 K) and hot (300 K) baths are set near the fixed positions to obtain the thermal conductivity at 300 K. After total relaxation of the system, the temperature profiles of silicene, germanene, and the Si-Ge lateral heterostructure are demonstrated by Figure 5b. For the pure silicene and germanene monolayers, the temperature gradient (*dT*/*dx*) is acquired through fitting temperature profiles. Next, thermal conductivity (*k*) is computed through the Fourier law:

(3) $$k=\frac{J}{AdT/dx}$$

where *A* means the cross-sectional area, through which the heat flux passes. In particular, five Si-Ge interfaces are built in the lateral heterostructure with equal length of silicone and germanene, shown in Figure 5a. Thus, the thermal conductivity of silicene, germanene, and the lateral heterostructure in the armchair direction (or zigzag direction) are obtained as 19.17 (or 22.45) W·m^−1^·K^−1^, 6.40 (or 7.53) W·m^−1^·K^−1^, and 3.09 (or 3.47) W·m^−1^·K^−1^, respectively. Obviously, the phonon dispersion at the interface significantly inhibits heat conductivity in the lateral heterostructure.

**Figure 6:**
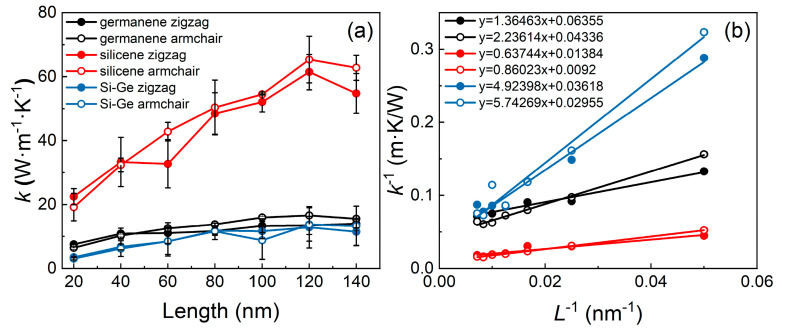
The relationship between (**a**) thermal conductivity and sample length, and (**b**) inverse thermal conductivity and inverse sample length of silicene, germanene, and the lateral heterostructure.

For the purpose of detecting the influence of size on the thermal attributes [55,56], the sample width is fixed as 5 nm with the length between 20 nm and 140 nm. As can be seen in Figure 6, the pure germanene and silicene monolayers exhibit isotropic thermal transport characteristics, and the longer monolayers have higher thermal conductivity. Evidently, the thermal conductivity of silicene exceeds that of germanene. Interestingly, the thermal conductivity of the Si-Ge heterostructure is between that of the silicene and germanene monolayers; when the length is over 100 nm, the obtained thermal conductivity of the Si-Ge heterostructure surpasses that of the two monolayers. Specifically, as the sample length ranges between 20 nm and 140 nm, the thermal conductivity of silicene extends from 22.45 W·m^−1^·K^−1^ to 54.76 W·m^−1^·K^−1^ in the zigzag direction, and from 19.17 W·m^−1^·K^−1^ to 62.76 W·m^−1^·K^−1^ along the armchair orientation. Furthermore, the thermal conductivity of germanene can be enhanced from 7.53 W·m^−1^·K^−1^ to 15.56 W·m^−1^·K^−1^ along the zigzag orientation, and from 6.41 W·m^−1^·K^−1^ to 15.56 W·m^−1^·K^−1^ along the armchair orientation. The thermal conductivity of Si-Ge heterostructures also exhibits significant increases between 3.47 W·m^−1^·K^−1^ and 11.47 W·m^−1^·K^−1^ along the zigzag orientation, and between 3.09 W·m^−1^·K^−1^ and 13.29 W·m^−1^·K^−1^ along the armchair orientation, shown in Figure 6a. The obtained thermal conductivity of Si-Ge heterostructures is also larger than that of the bulk Si-Ge-Si heterostructure (about 2–4 W·m^−1^·K^−1^) [57] and comparable with that of the doped Si-Ge heterostructure [58]. Moreover, the error bars in Figure 6a indicate that even though some individual thermal conductivity has decreased, the overall thermal conductivity of the monolayers and the Si-Ge heterostructure has increased by size. The thermal conductivity has a large temperature dependency when the phonon mean free path (MFP) exceeds the system length. To obtain an efficient MFP, the relationship between length and thermal conductivity is explored by the following equation [59]:

(4) $$k^{-1}=k_{\infty }^{-1}\left(\frac{l}{L}+1\right)$$

where $k_{\infty }^{-1}$ is the thermal conductivity for an infinite sample length and l is the MFP. From the aforementioned, as shown in Figure 6b, it can be established that the effective phonon mean free ranges of pure silicene and germanene are 36.52 nm and 69.78 nm, respectively, which are smaller than those of the zigzag and armchair heterostructures, which are 136.09 nm and 194.34 nm, respectively.

## 4. Conclusions

This work uses systematic molecular dynamics computations to examine in-plane mechanical and heat transport features of the Si-Ge lateral heterostructure. The influence of pre-existing fractures, temperature, and vacancy defects on the mechanical characteristics of the Si-Ge lateral heterostructure is investigated by applying uniaxial strain. It is found that the mechanical properties of germanene are key elements for determining the fracture strain and strength of Si-Ge heterostructures. The mechanical properties of the heterostructures can be effectively regulated by temperature and by introducing pre-existing cracks and vacancy defects. With the small pre-existing cracks and vacancy defects in the silicene, the failure of the heterostructure still preferentially occurs in the germanene region. In terms of the heat transport performance of the Si-Ge heterostructure, the non-equilibrium molecular dynamics results show that the effective phonon mean free ranges of zigzag and armchair heterostructures are 136.09 nm and 194.34 nm, separately. This difference in the phonon mean free path is capable of remarkably influencing heat transport performance of the Si-Ge heterostructure. When the system length is shorter than the aforementioned path, thermal conductivity shows a strong temperature dependence, which is more pronounced in silicene. Furthermore, it is observed that the thermal conductivities of pure silicene and germanene increase with the sample length at the nanoscale. These findings have significant implications for applying Si-Ge heterostructures in electronic and thermoelectronic devices.

## Data Availability

The data presented in this study are available upon request from the corresponding author.

## Supplementary Materials

The following supporting information can be downloaded at: https://www.mdpi.com/article/10.3390/molecules29163823/s1.
