# Supplementary material for: Mechanical and Lattice Thermal Properties of Si-Ge Lateral Heterostructures
Source: Molecules. 2024 Aug 12;29(16):3823. doi: 10.3390/molecules29163823 (PMC11357318; doi:10.3390/molecules29163823)
Supplement: Supplementary file 1 [file molecules-29-03823-s001.zip › molecules-3125118-supplementary.pdf]

**Supporting Information**

# Mechanical and Lattice Thermal Properties of Si-Ge Lateral Heterostructures

**Liuhuan Zhao [1], Lei Huang [2], Ke Wang [3], Weihua Mu [4,*], Qiong Wu [1], Zhen Ma [5] and Kai Ren [1,*]**

[1] School of Mechanical and Electronic Engineering, Nanjing Forestry University, Nanjing 210037, China
[2] School of Mechanical Engineering, Southeast University, Nanjing 211189, China
[3] School of Automation, Xi'an University of Posts & Telecommunications, Xi'an 710121, China
[4] Wenzhou Institute, University of Chinese Academy of Sciences, Wenzhou 325000, China
[5] School of Agricultural Engineering, Jiangsu University, Zhenjiang 212013, China
* Correspondence: muwh@ucas.ac.cn (W.M.); kairen@njfu.edu.cn (K.R.)

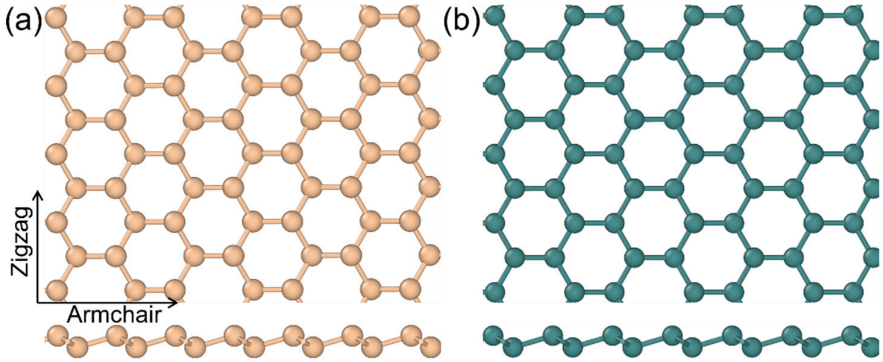


**Figure S1.** (**a**) is a schematic diagram of silicene and (**b**) is a schematic diagram of germanene.
